# Readiness for clinical practice: Self-perceived confidence of final year dental students in Turkey - A multi-institutional study

**DOI:** 10.12688/mep.20115.1

**Published:** 2024-03-08

**Authors:** Halenur Altan, Hakan Yasin Gönder, Elif Demirel, Ahmet Altan, Ali Rıza Tunçdemir, Daniel Zahra, Sadeq Ali Al Maweri, Kamran Ali

**Affiliations:** 1 Pediatric Dentistry Department, Faculty of Dentistry, Necmettin Erbakan University, Meram, Konya, Turkey; 2 Restorative Dentistry Department, Faculty of Dentistry, Necmettin Erbakan University, Meram, Konya, Turkey; 3 Maxillofacial Surgery Department, Faculty of Dentistry, Necmettin Erbakan University, Meram, Konya, Turkey; 4 Prosthdontics Department, Faculty of Dentistry, Necmettin Erbakan University, Meram, Konya, Turkey; 5 School of Psychology, University of Plymouth, Plymouth, England, PL4 8AA, UK; 6 College of Dental Medicine, Qatar University, Doha, Doha, 2713, Qatar

**Keywords:** Dental students, clinical practice, preparedness, undergraduate

## Abstract

**Introduction:**

The primary aim of undergraduate dental education is to prepare dental students for independent dental practice and to enable them to provide safe and effective dental care. This study aimed to investigate the self-perceived preparedness of senior dental undergraduate students in Turkey.

**Methods:**

Purposive sampling was used to recruit final-year dental students from 10 dental institutions offering undergraduate dental programs in Turkey. Student preparedness was assessed using a previously validated dental preparedness assessment scale based on 50 items encompassing core clinical skills, cognitive attributes, and behavioral skills. The research instrument was then translated into Turkish. The R statistical environment for Windows was used for the data analysis.

**Results:**

Responses were provided by 272 students (156 women and 116 men; 57% and 43%, respectively) across 10 different universities. The mean score of the participants was 75.68 with slightly higher scores for men compared to women (77.35 *vs.* 74.46 respectively). However, independent *t*-tests showed that the scores did not differ significantly between women and men.

**Conclusions:**

This study evaluated the self-perceived preparedness for dental practice of final-year students from 10 universities in Turkey. Although the results showed several areas of weakness, the scores of self-perceived preparedness of Turkish students were comparable to those reported in Europe and Asia. These findings can be used to inform future curriculum development to support students in consolidating their learning in perceived areas of weakness.

## Introduction

Undergraduate dental education aims to equip future dentists with scientific knowledge, evidence-based clinical skills, professionalism, communication skills, teamwork, reflective practice, and lifelong learning attitudes ^1, 2^. The ultimate goal of undergraduate dental education is to prepare dental students for dental practice to facilitate the smooth transition of dental graduates from a university setting into independent clinical practice ^3, 4^. Although dental schools provide education, training, and assessments for undergraduate students based on core learning outcomes, evidence from the literature shows that dental students may demonstrate gaps in their knowledge and skills at the time of graduation ^5– 9^. Therefore, it is important for dental schools to longitudinally assess the preparedness of their dental students throughout the undergraduate program to identify deficiencies in their knowledge and skills and provide early support and remediation ^10^.

Although formative and summative assessments of students’ performance in individual courses/modules at various stages of a dental program are fundamental to inform critical decisions regarding student progression, dental educators may also consider undertaking holistic evaluations of students’ preparedness benchmarked against program learning outcomes ^11^. Periodic evaluation of students’ preparedness for practice may provide an effective strategy to gauge the effectiveness of dental educational programs, teaching methods, curriculum implementation, and the professional development of students. By comparing individual student performance to benchmarks, dental educators can identify areas where students may require additional consolidation and make informed decisions to ensure that they are ready to provide safe and effective dental care independently by the time they graduate. Once students move away from the temporal confines of the university environment, they may find it more challenging to seek help to address their deficiencies ^4, 12^.

Undergraduate dental programs in Turkey span five years after high school education. The first three years focus on preclinical education and the didactic teaching of basic sciences and practical training in simulated dental learning environments. The last two years of education have been dedicated to training in clinical settings where students develop their skills in diagnosis and treatment planning, undertaking clinical procedures on patients, and demonstrating effective interpersonal skills, professionalism, and teamwork. Dental education in Turkey is regulated by the Council of Dental Deans Education and Research Subcommittee (CDD-ERS). Dental education providers are required to uphold contemporary standards of dental education to produce dental graduates who can deliver safe and evidence-based dental care to the community and are equipped with underpinning scientific knowledge, clinical skills, and comply with professional standards.

### Conceptual framework

The conceptual framework of the study was underpinned by the theory of situated learning, which views learning as a transformative process that is closely linked to the context and social interactions in learning environments ^13^. Dental students pursue their learning in multiple settings including lecture rooms, simulation laboratories, clinics, and community engagement activities. The students commence their journey at the periphery and develop their knowledge, skills, and professional values through active participation and assuming increasing social responsibility.

This study aimed to investigate the self-perceived preparedness of final-year undergraduate dental students in Turkey.

## Methods

### Research ethics

This study was approved by the Institutional Research Ethics Committee of Necmettin Erbakan University, Turkey (No. 2022/17-124, dated 28/04/2022). All participants provided informed written consent to participate in this study. All research data were processed and stored according to university data protection regulations.

### Study design

The study was a cross-sectional analytical study.

### Setting

The study was undertaken by the dental faculty at Necmettin Erbakan University, Turkey.

### Research instrument

A previously validated and widely used Dental Undergraduate Preparedness Assessment Scale (DU-PAS) was used for the data collection ^14, 15^. The DU-PAS is based on 50 items, including 24 related to clinical skills (Part A) and 26 related to scientific knowledge and affective skills (Part B).

Each item in Part A was scored on a three-point scale ranging from no experience (0), with verbal and/or practical input from a colleague (1), and independently (2). Items in part B of the DU-PAS are scored on a three-point scale: no experience (0), mostly (1), and always (2). The maximum score for the 50 items on the DU-PAS was 100.

### Participants

Participants were final year undergraduate dental students. The eligibility criteria were as follows:

- Over 18 years old
- Active student at one of the participating institutions
- Students who had interrupted their studies were not included in the study.

### Sampling technique

Purposive sampling was used to invite participants using institutional WhatsApp groups.

### Sample size calculation

In the adaptation of a scale to another culture, a group at least to 5–10 times larger than the number of scale items should be reached. In this study, the number of scale items was 50. In the validation of the DUPAS, the desired sample size was estimated to be at least 250 final-year undergraduate dental students, with at least five responses for each of the 50 items in the scale ^16^.

### Data collection

All participants provided informed consent to participate in the study online, and provided their responses to the DU-PAS independently on Google forms. The data collection was done from June 2022 to November 2022. Demographic data related to gender, year of study, and institution were also collected. All responses were recorded anonymously and it was not possible to identify individual participants.

### Data analysis

Data analysis was conducted using R statistical environment for Windows version 4.3.1 (R Core Team, 2022) ^17^. Descriptive statistics were calculated to describe the sample and subgroups and their distributions of scores for each part and between genders. Chi-square tests of association were conducted to compare the distribution of response options between groups where they were treated categorically, and independent *t*-tests were used where responses were treated as numeric. Pearson’s correlation coefficients were used to evaluate the relationships between the scores for the different items.

## Results

Responses were provided by 272 students (156 women and 116 men; 57% and 43%, respectively) ^15^ across 10 different universities, as shown in Table 1. All participants were Year 5 students. Missing data was replaced with 0 (‘No Experience’); this amounted to 11 individual responses across 11 different students (one missing data point for each of the 11 students, and mostly across different items).

**Table 1.** Proportions of student sample by university and gender.

	Frequency			Percentage		
University	Women	Men	Total	Women	Men	Total
Adiyaman	12	10	22	7.69	8.62	8.09
Akdeniz	7	4	11	4.49	3.45	4.04
Erciyes	22	7	29	14.10	6.03	10.66
Gaziantep	5	9	14	3.21	7.76	5.15
Harran	1	0	1	0.64	0.00	0.37
Hatay Mustafa Kemala	12	14	26	7.69	12.07	9.56
Kütahya saglik bilimleri	8	5	13	5.13	4.31	4.78
Ordu	1	0	1	0.64	0.00	0.37
Selçuk	10	3	13	6.41	2.59	4.78
Tokat Gaziosmanpasa	20	9	29	12.82	7.76	10.66
Missing	58	55	113	37.18	47.41	41.54
Total	156	116	273			

### Mean scores by item

Descriptive statistics by item (mean scores with standard deviation, 95% confidence intervals, and range) were computed. Table 2 summarizes the descriptive statistics for Part A with 24 items (n=24), and Table 3 summarizes the descriptive statistics for Part B with 26 items (n=26), in decreasing order. These were calculated by converting the categorical responses to numerical values (No Experience = 0, With Input/Mostly = 1, Independent/Always =2).

**Table 2.** Mean scores of participants for Part A (in decreasing order).

Item ‘I am able to…’	n	M	SD	U95CI	L95CI	Min	Max	Range	IQR
1. Obtain a complete medical history from my patients	272	1.93	0.30	1.97	1.90	0	2	2	0
11. Obtain a valid consent from my patients prior to undertaking any treatment.	272	1.93	0.28	1.96	1.90	0	2	2	0
16. Remove dental caries effectively	272	1.93	0.28	1.96	1.90	0	2	2	0
14. Administer inferior dental nerve blocks effectively	272	1.92	0.30	1.95	1.88	0	2	2	0
19. Perform endodontic treatment on single rooted teeth appropriately	272	1.91	0.31	1.95	1.87	0	2	2	0
4. Undertake periapical radiographs	272	1.90	0.35	1.94	1.86	0	2	2	0
17. Restore teeth with tooth-coloured fillings appropriately	272	1.88	0.37	1.93	1.84	0	2	2	0
12. Carry out patients’ treatment sessions in an appropriate order	272	1.81	0.42	1.86	1.75	0	2	2	0
15. Perform non-surgical periodontal treatment using appropriate methods	272	1.81	0.45	1.86	1.76	0	2	2	0
6. Interpret common findings on dental radiographs	272	1.80	0.42	1.85	1.75	0	2	2	0
24. Undertake non-surgical tooth extractions appropriately	272	1.79	0.42	1.84	1.74	0	2	2	0
2. Undertake a comprehensive, clinical oral examination	272	1.78	0.44	1.83	1.72	0	2	2	0
10. Explain the merits and demerits of various treatment options to my patients	272	1.73	0.46	1.78	1.67	0	2	2	1
3. Prescribe appropriate dental radiographs	272	1.68	0.49	1.73	1.62	0	2	2	1
21. Provide crowns using principles of tooth preservation	272	1.60	0.58	1.67	1.53	0	2	2	1
9. Provide a range of treatment options to my patients based on their individual circumstances	272	1.58	0.53	1.64	1.51	0	2	2	1
5. Undertake bitewing radiographs	272	1.48	0.82	1.58	1.38	0	2	2	1
8. Formulate a comprehensive treatment plan which addresses all treatment needs of my patients	272	1.39	0.53	1.45	1.33	0	2	2	1
23. Provide mechanically sound full dentures	272	1.38	0.61	1.45	1.30	0	2	2	1
20. Perform endodontic treatment on multi rooted teeth appropriately	272	1.37	0.68	1.45	1.29	0	2	2	1
22. Provide mechanically sound partial dentures	272	1.29	0.65	1.37	1.21	0	2	2	1
13. Prescribe drugs to my patients appropriately	272	1.18	0.58	1.25	1.11	0	2	2	1
7. Assess the treatment needs of patients requiring orthodontics	272	1.08	0.65	1.16	1.01	0	2	2	1
18. Restore teeth with amalgam fillings appropriately	272	0.88	0.91	0.99	0.77	0	2	2	2

**Table 3.** Mean scores of participants for Part B (in decreasing order).

Item	n	Mean	SD	U95CI	L95CI	Min	Max	Range	IQR
37. I provide opportunities for my patients to express their expectations from dental treatment	272	1.75	0.45	1.81	1.70	0	2	2	0
36. I feel I can manage to communicate effectively with my patients	272	1.70	0.47	1.75	1.64	0	2	2	1
40. I feel confident to communicate appropriately with my colleagues	272	1.69	0.49	1.75	1.63	0	2	2	1
27. I recognise my personal limitations in clinical practice	272	1.68	0.48	1.74	1.62	0	2	2	1
50. I take appropriate measures to protect patient confidentiality	272	1.64	0.53	1.70	1.57	0	2	2	1
38. I feel confident to address barriers to effective communication with patients appropriately	272	1.63	0.50	1.68	1.57	0	2	2	1
39. I feel confident to communicate potential risks of operative procedures to patients	272	1.59	0.51	1.65	1.53	0	2	2	1
43. I am able to fulfil my responsibilities as an effective member of the dental team	272	1.59	0.52	1.65	1.53	0	2	2	1
46. I take responsibility for my continuing professional development	272	1.57	0.54	1.64	1.51	0	2	2	1
26. I feel able to motivate my patients to encourage self-care for their dental needs	272	1.56	0.51	1.62	1.50	0	2	2	1
48. I restrict my relations with my patients to a professional level	272	1.55	0.51	1.61	1.49	0	2	2	1
31. I reflect on my clinical practice in order to address my learning needs	272	1.54	0.53	1.61	1.48	0	2	2	1
41. I feel confident managing anxious patients with appropriate behavioural techniques	272	1.52	0.53	1.58	1.46	0	2	2	1
28. I feel comfortable asking for help from supervisor or colleague if needed	272	1.50	0.57	1.57	1.44	0	2	2	1
25. I feel I can manage peoples’ expectations of their treatment	272	1.38	0.52	1.44	1.31	0	2	2	1
44. I maintain accurate records of my clinical notes	272	1.38	0.57	1.45	1.31	0	2	2	1
47. I am aware of my legal responsibilities as a dental professional	272	1.38	0.62	1.45	1.31	0	2	2	1
45. I am able to work within the constraints of clinical appointment schedules	272	1.37	0.58	1.44	1.30	0	2	2	1
42. I am able to manage the behaviour of children to enable appropriate dental treatment	272	1.30	0.51	1.36	1.24	0	2	2	1
29. I am able to refer patients with complex treatment needs appropriately	272	1.29	0.62	1.36	1.21	0	2	2	1
49. I feel able to raise concerns about inappropriate behaviour of my colleagues	272	1.27	0.67	1.35	1.19	0	2	2	1
35. I use an evidence-informed approach in my clinical practice.	272	1.19	0.60	1.26	1.12	0	2	2	1
32. I have sufficient knowledge of scientific principles which underpin my dental practice	272	1.11	0.52	1.18	1.05	0	2	2	0
34. I am confident to interpret the results of research which may influence my practice	272	1.03	0.65	1.11	0.95	0	2	2	0
33. I am confident to evaluate new dental materials and products using an evidence-based approach	272	0.86	0.69	0.94	0.78	0	2	2	1
30. I feel confident referring patients with suspected oral cancer	272	0.67	0.74	0.76	0.58	0	2	2	1

### Overall scores

The overall scores by part (A and B) and gender (women and men) are shown in Table 4. Independent *t*-tests did not show any significant differences between women and men in Part A ( *t*_(224.10)_=-1.80, *p*=0.07), Part B ( *t*_(261.00)_=-1.30, *p*=0.20), or overall ( *t*_(206.67)_=-1.80, *p*=0.07).

**Table 4.** Descriptive statistics for overall scores by part (A and B) and gender.

Part	Gender	n	Mean	SD	Min.	Max.	Range	IQR
A (24 Items)	Women	156	38.45	5.68	0	48	48	6.00
	Men	116	39.83	6.59	0	48	48	8.00
	Combined	272	39.03	6.11	0	48	48	7.00
B (26 Items)	Women	156	36.15	8.45	11	52	41	12.25
	Male	116	37.56	8.99	3	52	49	12.00
	Combined	272	36.75	8.69	3	52	49	12.00
Overall (50 Items)	Women	156	74.46	11.35	37	98	61	15.50
	Men	116	77.35	13.78	3	100	97	16.00
	Combined	272	75.68	12.49	3	100	97	16.00

### Comparison by gender

**Figure 1.**
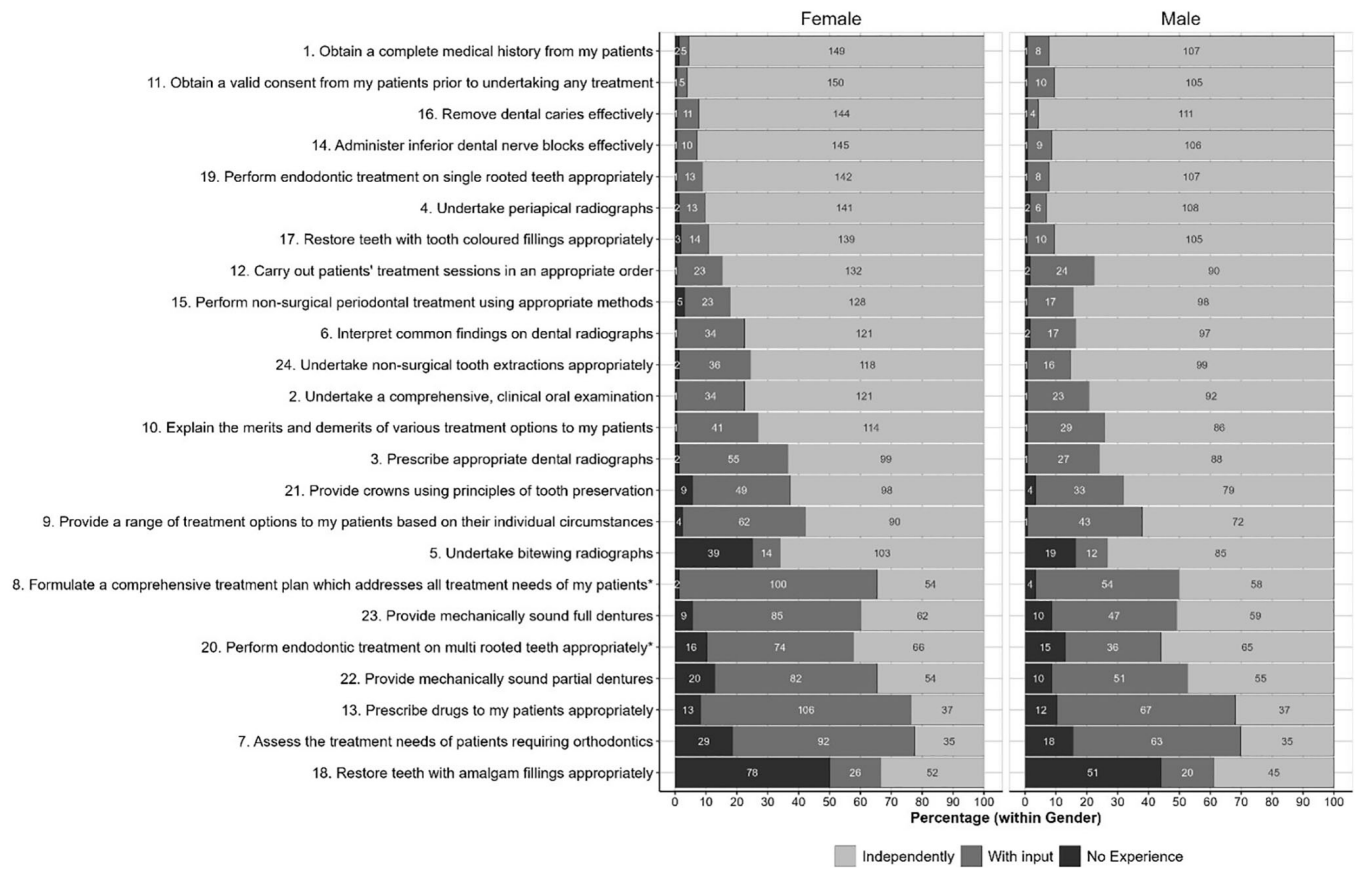
Distributions of scores for each item in Part A given by women and men. Starred items are items for which Chi-squared tests shows a statistically significant variation in the distributions between genders at the p<0.05 level.

**Figure 2.**
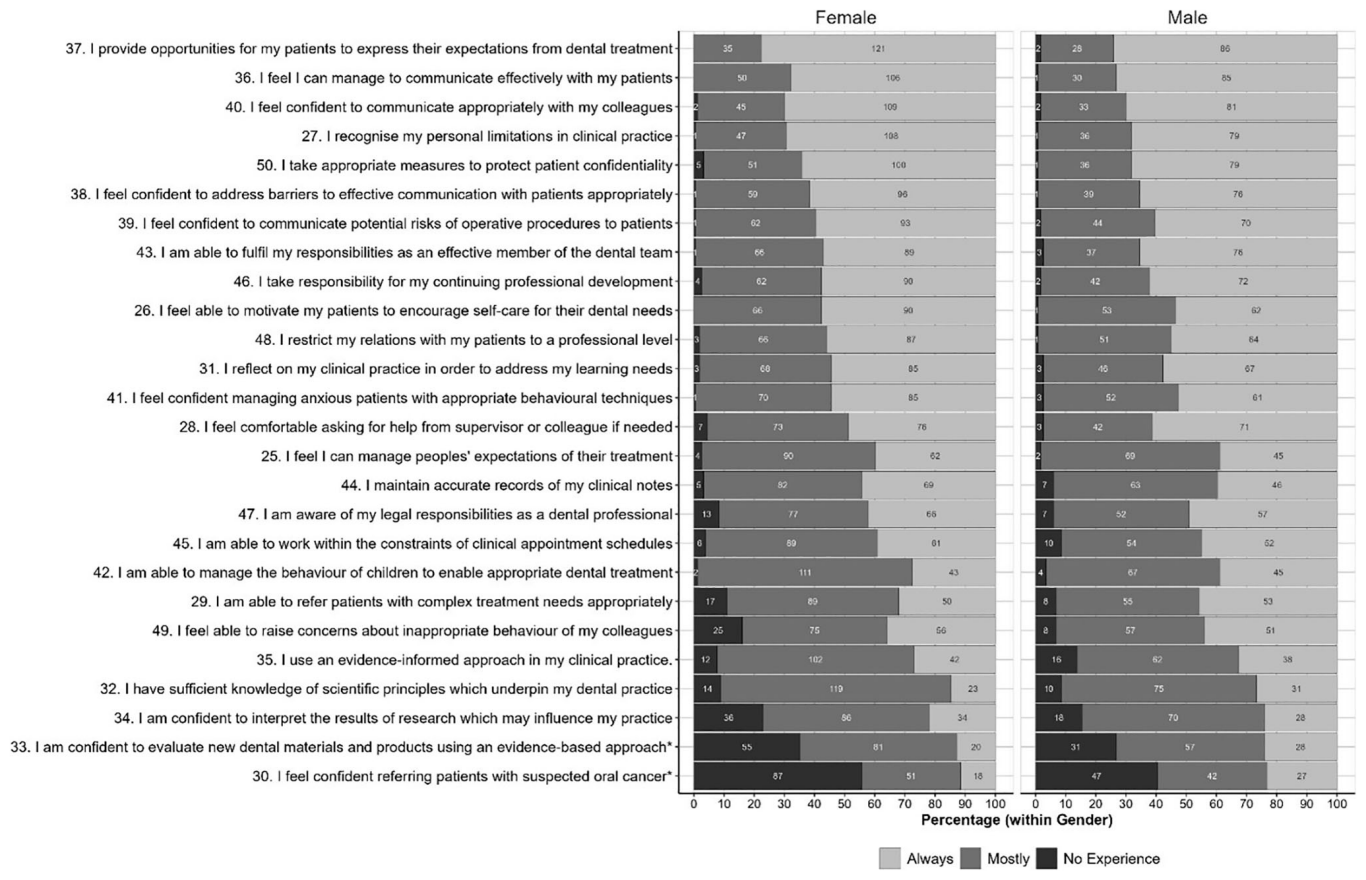
Distributions of scores for each item in Part 2 given by women and men. Starred items are items for which Chi-squared tests shows a statistically significant variation in the distributions between genders at the p<0.05 level.

Small numbers of students from some universities and uniformity of stage of study preclude any meaningful analysis of these factors, although a comparison of student scores by gender is shown for Part A in Figure 1 and Part B in Figure 2. The starred items are items for which chi-squared tests show a statistically significant variation in the distributions between genders at the *p*<0.05, although very few of the items in each part showed statistically significant differences between genders; only two in each part.

### Correlations between items

**Figure 3.**
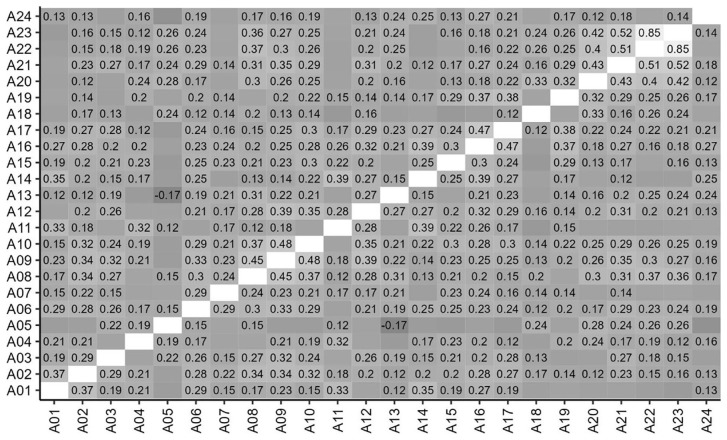
Inter-Item correlations of student scores for Part A items. Correlations coefficients are shown for those that are statistically significant at p<0.05.

**Figure 4.**
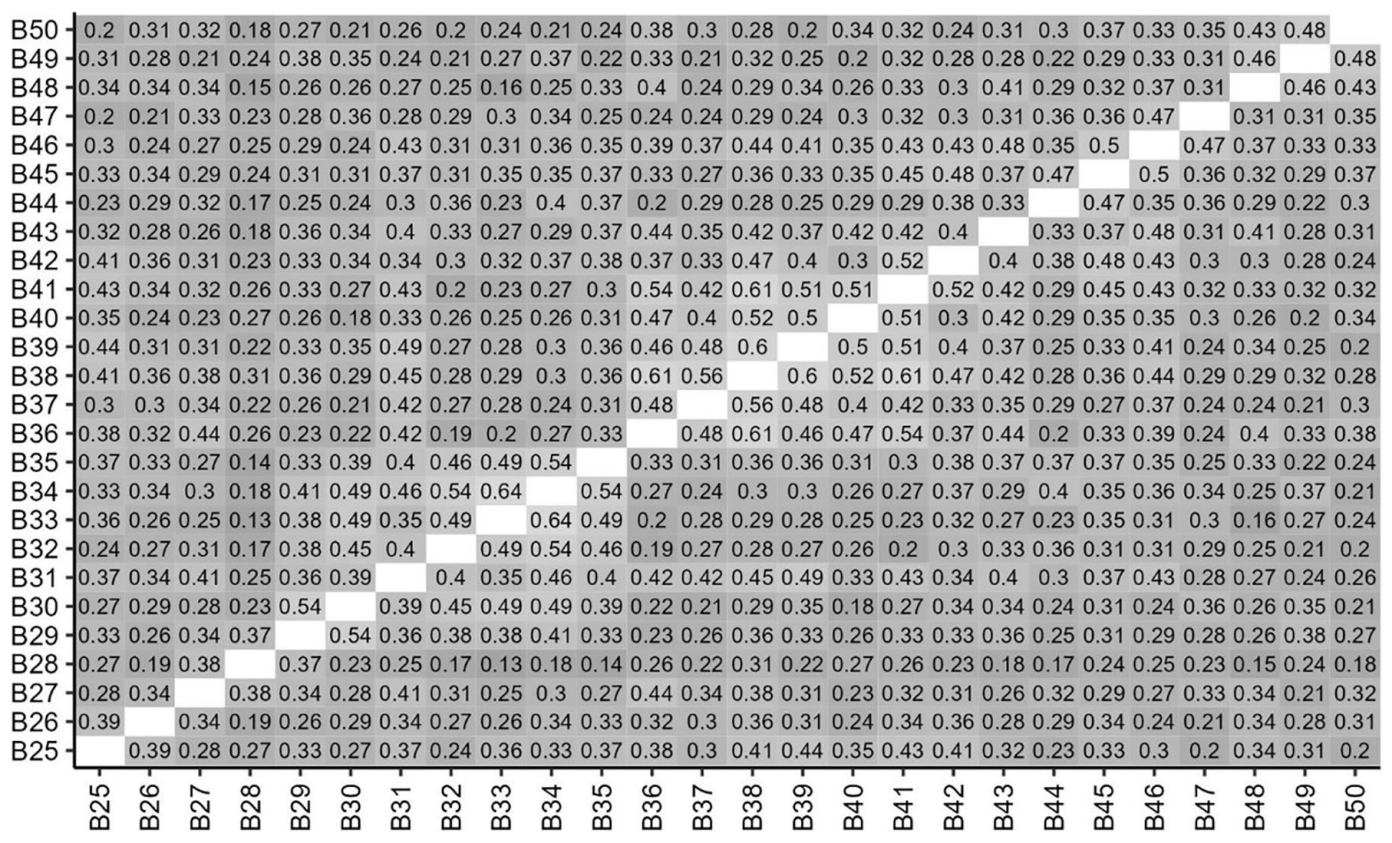
Inter-item Correlations of student scores for Part B items. Correlations coefficients are shown for those that are statistically significant at p<0.05.

Figure 3 and Figure 4 show the correlations between ratings for items in Parts A and B. Correlation coefficients are shown for those that are statistically significant at *p*<0.05, and stronger, positive correlations are shown in lighter shades. It can be seen that scores were significantly and positively correlated for the majority of Part A items and all Part B items.

### Discussion

The DU-PAS is a comprehensive scale used to evaluate the preparedness of undergraduate dental students and new graduates. It encompasses core clinical skills, scientific knowledge, and affective attributes, including communication skills, professionalism, and interpretation of research evidence. To the best of our knowledge, this is the first study to evaluate the preparedness of final-year undergraduate dental students in Turkey.

Numerous studies have utilized the DU-PAS to assess the preparedness of dental students and new graduates in different countries ^18– 21^. The DU-PAS scores of the self-perceived assessment reported by the student participants in this study were comparable to those reported in the aforementioned studies. In the present study, the mean scores of self-perceived preparedness of final-year dental students were 75.68%, which was higher than the mean score reported by final-year dental students in Pakistan (61.1%) ^21^ and the United Kingdom (74%) ^18^, but slightly lower than that of students in Malaysia (79.5%) ^20^. Independent *t*-tests did not reveal any significant differences between women and men, either overall or within Part A or Part B separately.

 The main limitation of this study is that self-assessment may not truly reflect the actual preparedness of students and new graduates. Previous studies on students in healthcare professions have shown poor correlations between their self-perceived competence and assessment by their supervisors, and students usually tend to score themselves higher than their supervisors ^22– 24^. Notwithstanding the potential for the inflation of self-assessment scores, several weaknesses in the preparedness of final-year students were identified. Gaps in the preparedness of students certainly need further investigation so that they can be addressed prior to graduation. The findings may be used by participating institutions to inform future curriculum development and identify further opportunities to enhance the learning experiences of their students to support their preparedness for practice after graduation.

The results identified several weaknesses related to clinical skills, as suggested by participants’ self-perceived confidence. The participants felt the least confident in restoring their teeth with amalgam fillings. However, this is not surprising because in recent years, there has been a decreasing trend in the use of amalgam in clinical dental practice and dental institutions in many countries, including Turkey, which places less emphasis on amalgam restorations ^25^. In contrast, the majority of the participants reported being confident in restoring teeth with tooth-colored filling materials, given the trends in contemporary clinical dentistry. A lack of confidence in providing amalgam fillings is indicative of changing clinical practice and does not appear to be a source of concern. However, low scores for confidence in performing endodontics on multi-rooted teeth are a source of concern. Multi-rooted endodontics appears to be challenging for undergraduate dental students, and similar findings have been reported from dental institutions across the board ^3, 8, 18– 21^. A number of factors may be responsible for the challenges in performing endodontics on multirooted teeth, including complex anatomy and inadequate access, which may lead to difficulties in the application of rubber dams and undertaking radiographs, as well as the need to access, prepare, and obtain multiple canals in posterior teeth. These challenges may adversely influence student confidence and warrant appropriate measures to support students in consolidating their endodontic skills. Additional learning opportunities in simulated dental learning environments and enhanced clinical exposure to multirooted teeth in endodontic clinics. In addition, dental students need structured teaching regarding the effective use of modern technology, which may facilitate endodontic diagnosis and treatment ^26, 27^.

Confidence in the assessment of orthodontic needs of patients was also identified as a weakness, and the findings corroborate those of other studies on the preparedness of dental students ^18, 19, 28^. Marked variations in undergraduate orthodontic curricula have been reported not only at a global level but also in individual countries with institutions working under a single regulator ^29^. Given that orthodontics is a specialized field, teaching at the undergraduate level needs to focus primarily on orthodontic assessment for a timely referral to specialist orthodontists ^30^.

 Low confidence was reported by the participants regarding their ability to appropriately prescribe medications. Prescription of analgescis, antimicrobials, and variety of locally applied medications is a common practice in clinical dentistry and dental students need adequate teaching on this core skill. Inadequate prescribing skills among medical and dental students have been widely reported in the literature ^31– 33^. Although dental institutions provide courses in pharmacology, teaching is usually delivered in the early years as part of the basic sciences by medical colleagues. Consequently, dental students are unable to contextualize their knowledge of clinical practice and may not retain their knowledge when they progress to clinical years ^34^. Dental educators need to explore new approaches to teach drug prescriptions, including case-based learning, role-play, and close supervision, to guide students in drug prescriptions in clinical practice.

Although the participants in the current study appeared to be confident in undertaking periapical radiographs, their confidence was reported to be low for bitewing radiographs. This gap appears to be related to the less frequent use of bitewing radiographs in Turkish dental institutions, which mainly rely on periapical radiographs for the diagnosis of common dental diseases. Similar findings have also been reported in other developing countries ^19, 21^. It is recognized that bitewing radiographs are the most appropriate for diagnosing interproximal caries, and dental institutions in Turkey need to revisit their teaching on dental radiography to address this gap. Finally, confidence in the provision of removable prostheses, including partial and full dentures, was reported to be low. Removable prosthodontic education seems to be a common challenge for dental institutions globally because of the limited availability of suitable patients, which restricts the clinical experience of undergraduate students ^35, 36^. This has prompted dental educators to use innovative methods to deliver prosthodontic education, including video-based learning and game-based trainings ^37, 38^. Moreover, dental institutions may collaborate with dental practitioners in primary care settings to improve the availability of suitable patients and ensure adequate clinical experience for dental students.

The main areas of weakness for attributes in Part B of the DU-PAS were related to the use of an evidence-based approach in clinical practice, the evaluation of new dental materials and products, and the interpretation of research findings. Skills in the interpretation of research findings and the use of evidence-based approaches to inform clinical practice are being recognized as increasingly important in clinical dentistry in the modern era ^39– 41^. However, the knowledge and skills of students to interpret research and use it effectively to support their clinical practice require further improvement ^42^. Didactic research courses do not appear to be the most effective method for imparting research skills to dental students. A multifaceted approach is suggested when teaching research skills and evidence-based dentistry with formal training in the critical appraisal of literature and the effective use of modern technology incorporating mobile devices, simulation, and online resources ^43^.

Referral for suspected oral cancer (OC) was also highlighted as an area of concern. OC is a significant global public health issue, and early detection is critical for favorable treatment outcomes and improved survival rates ^44, 45^. Dental professionals can play a critical role in the fight against OC by contributing to the prevention, early detection, and prompt referral of suspected OC to specialists ^46^. Previous studies on dental students and graduates have also highlighted gaps in the recognition and referral of OC ^18– 20^. The common factors underlying lack of confidence in the detection and referral of OC among dental students relate to insufficient training and exposure to suspected OC. The findings of this study underscore the need for revisting the teaching and training of oral cancer in undergraduate dental programs. There is a need to provide structured learning opportunities to dental students in specialist clinical settings, where they can observe the diagnosis and management of suspected oral cancer patients ^47^.

In summary, the findings of this study have highlighted several areas of weakness in the preparedness of dental students in Turkey, and similar gaps have been reported in studies on dental students from Europe, Asia, and Australia. Despite the limitations of self-assessment, encouraging students to evaluate their preparedness for dental practice provides them with opportunities to evaluate their performance holistically against program learning outcomes. It also encourages them to look ahead to appreciate what is expected from a dental graduate, inculcating a sense of professional identity ^48, 49^. Addressing the gaps in undergraduate dental education is essential to ensure that future dental graduates are ready for independent clinical practice and are able to deliver safe and effective dental care to the community. The findings of the current study may be used to inform the further development of undergraduate dental curricula to enhance dental graduates’ preparedness.

### Conclusions

This study evaluated the self-perceived preparedness for dental practice of final-year students from 10 universities in Turkey. Although several areas of weakness were identified, Turkish students’ self-perceived preparedness scores were comparable to those reported in Europe and Asia. These findings can be used to inform future curriculum development to support students in consolidating their learning in perceived areas of weakness.

## Data Availability

OSF: Preparedness of Dental Students Data File. https://doi.org/10.17605/OSF.IO/UHMYT ^15^. The project contains the following underlying data: DU-PAS Turkey Anon.xlsx OSF: Preparedness of Dental Students Data File. https://doi.org/10.17605/OSF.IO/UHMYT ^15^. The project contains the following extended data: Dental undergrad preparedness assessment scale DU-PAS.pdf Data are available under the terms of the Creative Commons Attribution 4.0 International license (CC-BY 4.0).
