# Supplementary material for: Readiness for clinical practice: Self-perceived confidence of final year dental students in Turkey - A multi-institutional study
Source: MedEdPublish (2016). 2024 Jul 11;14:9. Originally published 2024 Mar 8. [Version 2] doi: 10.12688/mep.20115.2 (PMC11306955; doi:10.12688/mep.20115.2)
Supplement: Peer review history

# Reviewer report: Reviewer response for version 2

**DOI:** 10.21956/mep.21936.r38217

**Published:** 2024-08-07

**Authors:** Muhammad Haseeb

**Affiliations:** 1 The University of Lahore, Lahore, Punjab, Pakistan

Manuscript is well written and the study design is appropriate and allows replication by others. Statistical tests performed are also appropriate and address the objectives of the study. Discussion is eloquently written with relevant articles cited. Limitations of the study was addressed and rationally justified. Conclusion is supported by the results. 

 Manuscript is hence approved.

Have any limitations of the research been acknowledged?

Yes

Is the work clearly and accurately presented and does it cite the current literature?

Yes

If applicable, is the statistical analysis and its interpretation appropriate?

Yes

Are all the source data underlying the results available to ensure full reproducibility?

Yes

Is the study design appropriate and is the work technically sound?

Yes

Are the conclusions drawn adequately supported by the results?

Yes

Are sufficient details of methods and analysis provided to allow replication by others?

Yes

Reviewer Expertise:

Dentistry, Medical education, Work-placed based assessments, Mentoring.

I confirm that I have read this submission and believe that I have an appropriate level of expertise to confirm that it is of an acceptable scientific standard.

# Reviewer report: Reviewer response for version 2

**DOI:** 10.21956/mep.21936.r37912

**Published:** 2024-07-12

**Authors:** Wen Wu Tan

**Affiliations:** 1 Asian Institute of Medicine Science and Technology University, Bedong, Kedah, Malaysia

The authors have addressed the concerns raised.

Have any limitations of the research been acknowledged?

Partly

Is the work clearly and accurately presented and does it cite the current literature?

Yes

If applicable, is the statistical analysis and its interpretation appropriate?

Yes

Are all the source data underlying the results available to ensure full reproducibility?

Yes

Is the study design appropriate and is the work technically sound?

Yes

Are the conclusions drawn adequately supported by the results?

Partly

Are sufficient details of methods and analysis provided to allow replication by others?

Partly

Reviewer Expertise:

Dental education, cross-sectional studies

I confirm that I have read this submission and believe that I have an appropriate level of expertise to confirm that it is of an acceptable scientific standard.

# Reviewer report: Reviewer response for version 1

**DOI:** 10.21956/mep.21540.r37225

**Published:** 2024-06-08

**Authors:** Wen Wu Tan

**Affiliations:** 1 Asian Institute of Medicine Science and Technology University, Bedong, Kedah, Malaysia

**Overall comment**

 The overall manuscript is satisfactory **. **It contribute valuable information to the literature on the preparedness level of final-year dental students in Turkey. However, some modifications and polishing are needed before the paper can be accepted.

**Abstract**

 The abstract is concise and effectively summarizes the key points of the study.

**Introduction**

 The introduction is well-written and provides a clear overview of the topic. It also effectively justifies the necessity of conducting this research.

**Methods**

 There are too many subheadings under the Methods section. I suggest the authors combine them. For instance, 'Study Design,' 'Setting,' 'Research Ethics,' 'Participants,' 'Sampling Technique,' and 'Sample Size Calculation' can all be grouped under the heading 'Study Design and Participant Recruitment.'

 Additionally, the inclusion criteria need clarification. How do you define an active student? How do you determine if a student has interrupted studies? Please clarify these points and revise the inclusion criteria accordingly.

 When the authors mentioned that purposive sampling is used, do you mean that you specifically select certain groups of students to answer the survey? Since in the inclusion criteria it is mentioned that only active students with uninterrupted studies are chosen (the better students), they will naturally demonstrate better preparedness, resulting in biased outcomes, compared to the weaker students.

**Results**

 May I know what is the rationale of having ‘min, max, range and IQR’ in Table 2,3 & 4, as it seems redundant to me. You may not need so many different types of measures of dispersion in a table.

 I notice that the results are shown both as percentages and scores in two separate tables. Typically, presenting just one of these formats is sufficient.

**Discussion**

 This sentence appears to be incomplete. ‘Additional learning opportunities in simulated dental learning environments and enhanced clinical exposure to multirooted teeth in endodontic clinics’.

 Justification is not given as to why participants have low confidence in the assessment of orthodontic needs of patients.

 Please mention other limitations apart from self-assessment. Also, you may give recommendation on the direction of future research of similar topic.

Have any limitations of the research been acknowledged?

Partly

Is the work clearly and accurately presented and does it cite the current literature?

Yes

If applicable, is the statistical analysis and its interpretation appropriate?

Yes

Are all the source data underlying the results available to ensure full reproducibility?

Yes

Is the study design appropriate and is the work technically sound?

Yes

Are the conclusions drawn adequately supported by the results?

Partly

Are sufficient details of methods and analysis provided to allow replication by others?

Partly

Reviewer Expertise:

Dental education, cross-sectional studies

I confirm that I have read this submission and believe that I have an appropriate level of expertise to confirm that it is of an acceptable scientific standard, however I have significant reservations, as outlined above.
